# Birdshot Chorioretinopathy in a Patient on Tocilizumab for the Treatment of Giant Cell Arteritis

**DOI:** 10.1155/crop/4861600

**Published:** 2025-10-10

**Authors:** Joshua Pasol, Elena B. Roth, Thomas A. Albini

**Affiliations:** Department of Ophthalmology, Bascom Palmer Eye Institute University of Miami Miller School of Medicine, Miami, Florida, USA

**Keywords:** birdshot chorioretinopathy, giant cell arteritis, tocilizumab

## Abstract

Birdshot chorioretinopathy (BSCR) is a presumed autoimmune disease of the eye affecting mostly middle-aged women and typically associated with the HLA-A29 genetic haplotype. Presenting symptoms include blurred vision, nyctalopia, and floaters. Classic fundus findings are multiple, bilateral, creamy, oval lesions around the optic nerve, mostly nasal and inferior to the disc. Fluorescein/indocyanine angiography, optical coherence topography, and fundus autofluorescence can be used for diagnosis and disease monitoring. Here, we present a case of a patient with giant cell arteritis (GCA) on tocilizumab who presented with asymptomatic BSCR lesions in both eyes. Both GCA and BSCR have T cell lymphocyte–mediated inflammation and are treated with corticosteroids. In our case, the patient was on a steroid-sparing agent, which may have suppressed symptoms of BSCR. We found no other cases of a patient having both GCA and BSCR.

## 1. Case Presentation

A 65-year-old Caucasian female was noted to have incidental, bilateral, chorioretinal lesions in both eyes during a follow-up exam for a prior history of giant cell arteritis (GCA). She had a history of biopsy-proven GCA at age 57 diagnosed after complaints of jaw claudication and myalgias treated with 3 years of oral prednisone and twice monthly tocilizumab, which was started at age 58. At age 61, the patient presented with ischemic optic neuropathy in the left eye despite a lack of GCA symptoms; however, the sedimentation rate was elevated at 85 mmg/h (C-reactive protein was normal at < 0.1 mg/dL). She was placed on prednisone 80 mg, and a repeat temporal artery biopsy was performed, which was positive. The GCA was controlled with tapering doses of prednisone and weekly tocilizumab.

**Figure 1:**
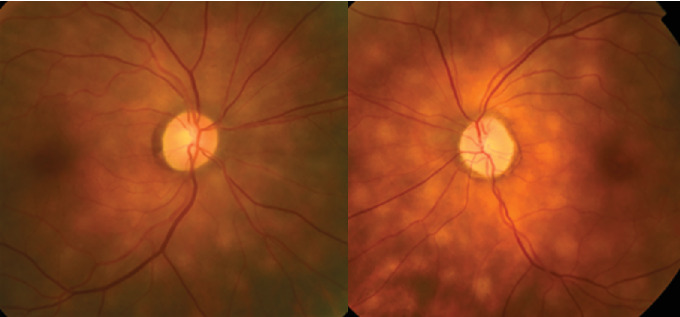
Fundus photos of both eyes identifying the mostly peripapillary birdshot lesions. The lesions appear round to oval in shape and have a creamy yellow–white appearance.

**Figure 2:**
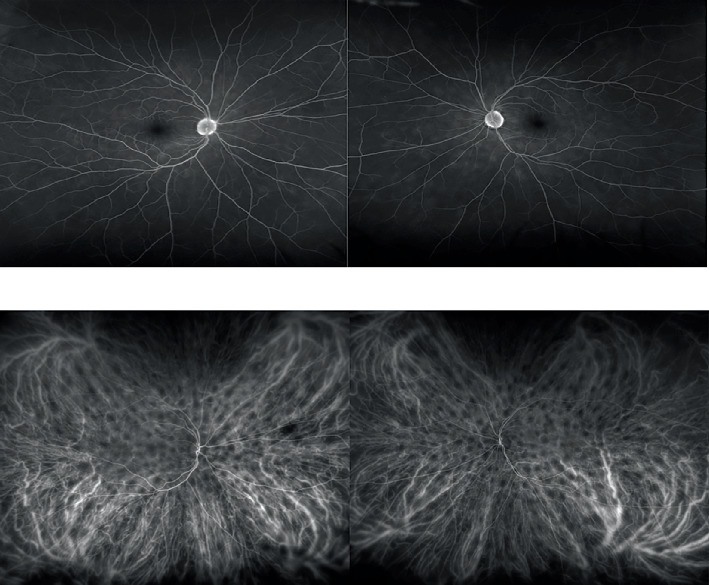
(a) Fluorescein angiogram (FA) and (b) indocyanine green angiography (ICG). The FA shows late nerve staining in both eyes, some peripheral vascular leakage, and choroidal infiltrates. The ICG shows the classic hypofluorescent dark spots in both eyes in the mid phase that persist into the late phase of the angiogram.

Examination upon new chorioretinal findings revealed the vision to be 20/20 OU; color vision was normal OU with a left afferent pupillary defect. The anterior exam was significant for prior refractive surgery and cataracts OU without signs of anterior chamber inflammation. The posterior pole revealed mild pallor of the left optic nerve and new, bilateral, multiple, roundish, hypopigmented lesions in the choroid (Figure 1). The patient was referred to our uveitis specialist. She underwent fluorescein/indocyanine green angiography (Figure 2), retinal optical coherence tomography, B-scan ultrasonography, and lab testing for HLA-A29, angiotensin converting enzyme (ACE), and RPR, which were all negative. The diagnosis of birdshot chorioretinopathy (BSCR) was made based on the clinical exam, although the HLA-A29 was negative. Magnetic resonance imaging of the brain with and without contrast showed no evidence of malignancy. The patient remains visually stable and asymptomatic now 24 months post-BSCR diagnosis.

## 2. Discussion

BSCR is a presumed autoimmune disease of the eye affecting mostly the middle-aged, with women slightly more affected than men [1]. The term birdshot was first introduced in 1980 in 13 cases of multiple, creamy choroidal dots that resembled birdshot ammunition from a shotgun [2]. Presenting symptoms include blurred vision, nyctalopia, and floaters [1]. Examination findings are typically limited to the posterior pole without anterior chamber reaction [1]. Classic fundus findings are multiple, bilateral, creamy, oval lesions around the optic nerve, mostly nasal and inferior to the disc [1]. Fluorescein/indocyanine angiography, OCT, and fundus autofluorescence can be used for disease monitoring. The mechanism of BSCR is not fully understood, but there is an association with HLA-A29 that suggests a T cell–mediated process [1]. HLA-A29 was reported to be elevated in over 95% of cases of BSCR, but a negative test does not exclude the diagnosis [3]. Treatments include observation, steroids or immunosuppressants, or biologic agents [1].

GCA is an inflammatory disease that has also been linked to T cell activation [4]. Some patients with GCA also have a genetic risk of having an HLA-DRB1⁣^∗^04 allele [5]. This allele has also been linked to other autoimmune diseases such as rheumatoid arthritis and Type 1 diabetes [6, 7]. HLA genes are encoded on the short arm of chromosome 6, which encodes surface antigens expressed in cells [8]. HLA classes are divided into Class I, II, and III. Class I includes HLA-A, B, and C, which provide foreign peptides to CD8+ T cells, while the Class II HLA-DR, HLA-DQ, and HLA-DP provide peptides to CD4+ T cells [8]. Both GCA and BSCR can have a genetic predisposition for T cell–mediated autoimmunity. In our case, the HLA-A29 was negative but did not rule out the clinical diagnosis of BSCR.

Both GCA and BSCR can be treated with steroids and steroid-sparing immune modulators. Steroid-sparing agents used to treat GCA include methotrexate and tocilizumab, among others [9]. Tocilizumab has FDA approval for the treatment of GCA, which has also been used in some isolated cases of BSCR [10]. Other authors have reported the use of tocilizumab in other ocular inflammatory conditions [11]. Tocilizumab works by blocking the IL-6 receptors, which are found on the surface of leukocytes, among other locations. By blocking IL-6, a proinflammatory cytokine, there is a reduction in the inflammatory cascade [12].

It is possible that our patient did not experience any new visual symptoms due to BSCR since she was already receiving treatment with tocilizumab, which may have suppressed the BSCR symptomatology. Other causes of choroidal lesions were excluded in our case, such as sarcoid, syphilis, and lymphoma. Although GCA may present with choroidal ischemia, we did not see evidence of this on our FA/ICG [13]. Our patient has still been stable visually for over 2 years while remaining on tocilizumab alone. We found no other cases of a patient having both GCA and BSCR, rendering this case unique, and we do not know why she developed two distinct inflammatory conditions. This case underscores the importance of routine follow-up and the possible existence of multiple inflammatory diseases.

## Data Availability

Data sharing is not applicable to this article as no datasets were generated or analyzed during the current study.

## References

[1] Bousquet E., Duraffour P., Debillon L., Somisetty S., Monnet D., Brézin A. P. (2022). Birdshot Chorioretinopathy: A Review. *Journal of Clinical Medicine*.

[2] Ryan S. J., Maumenee A. E. (1980). Birdshot Retinochoroidopathy. *American Journal of Ophthalmology*.

[3] Zucchiatti I., Miserocchi E., Sacconi R., Bandello F., Modorati G. (2013). HLA-A29-Positive Uveitis: Birdshot Chorioretinopathy, What Else?. *Case Reports in Ophthalmology*.

[4] Koster M. J., Warrington K. J. (2017). Giant Cell Arteritis: Pathogenic Mechanisms and New Potential Therapeutic Targets. *BMC Rheumatology*.

[5] Gonzalez-Gay M. A. (2001). Genetic Epidemiology. Giant Cell Arteritis and Polymyalgia Rheumatica. *Arthritis Research*.

[6] Wysocki T., Olesińska M., Paradowska-Gorycka A. (2020). Current Understanding of an Emerging Role of HLA-DRB1 Gene in Rheumatoid Arthritis-From Research to Clinical Practice. *Cells*.

[7] Caramalho I., Matoso P., Ligeiro D. (2024). The Rare DRB1^∗^04: 08-DQ8 Haplotype Is the Main HLA Class II Genetic Driver and Discriminative Factor of Early-Onset Type 1 Diabetes in the Portuguese Population. *Frontiers in Immunology*.

[8] de Bakker P. I., Raychaudhuri S. (2012). Interrogating the Major Histocompatibility Complex With High-Throughput Genomics. *Human Molecular Genetics*.

[9] Castañeda S., Prieto-Peña D., Vicente-Rabaneda E. F. (2022). Advances in the Treatment of Giant Cell Arteritis. *Journal of Clinical Medicine*.

[10] Leclercq M., Le Besnerais M., Langlois V. (2018). Tocilizumab for the Treatment of Birdshot Uveitis That Failed Interferon Alpha and Anti-Tumor Necrosis Factor-Alpha Therapy: Two Cases Report and Literature Review. *Clinical Rheumatology*.

[11] Atienza-Mateo B., Prieto-Peña D., Vicente-Rabaneda E. F., Blanco R., González-Gay M. A., Castañeda S. (2022). Utility of Tocilizumab in Autoimmune Eye Diseases. *Expert Opinion on Biological Therapy*.

[12] Okuda Y. (2008). Review of Tocilizumab in the Treatment of Rheumatoid Arthritis. *Biologics: Targets and Therapy*.

[13] Casella A. M. B., Mansour A. M., EC S. (2022). Choroidal Ischemia as One Cardinal Sign in Giant Cell Arteritis. *International Journal of Retina and Vitreous*.

